# Recent Advances in Therapeutic Strategies for the Treatment of Pediatric Diseases

**DOI:** 10.3390/pharmaceutics17080954

**Published:** 2025-07-24

**Authors:** Peppino Mirabelli, Mariaevelina Alfieri

**Affiliations:** 1 Clinical and Translational Research Unit, Santobono-Pausilipon Children’s Hospital, 80129 Naples, Italy; p.mirabelli@santobonopausilipon.it; 2 Clinical Pathology, Santobono-Pausilipon Children’s Hospital, 80129 Naples, Italy

Medical research has been traditionally focused on diseases that primarily affect adults, often due to their greater impact on life expectancy and economic productivity. However, in recent years, there has been growing recognition of the importance of pediatric diseases. These conditions often differ significantly from those in adults in terms of causes and underlying mechanisms, necessitating more specialized and complex research approaches. Moreover, enrolling children in clinical trials can be particularly challenging, requiring tailored methodologies and the involvement of specially trained personnel.

Pediatric diseases also have a significant impact on public health. Indeed, beyond the medical aspects, pediatric diseases often place a significant emotional and practical burden on families. When a child becomes ill (especially in the case of chronic or life-threatening disease), parents or caregivers may need to take extended time off work or even leave their jobs entirely to provide care, leading to financial strain and disruption of daily life. Despite this, medical research on pediatric diseases remains less common than that on adult conditions.

More importantly, the research on pediatric diseases can lead to the development of new treatments and cures for diseases that affect children.

In recent years, gene therapy, biological drugs, and innovative treatments are opening new opportunities for diagnosis and treatment of children affected by different diseases such as immunodeficiencies, tumors, hematological disorders, and genetic diseases. Numerous studies on pediatric diseases have been initiated, and many of them have led to important advances in the understanding and treatment of these diseases.

Some drugs, such as biologicals and gene therapy, are changing the natural history and long-term fate of patients who until a few years ago were considered incurable. Precision medicine allows us to make individualized diagnoses and to guarantee increasingly personalized therapies: not only the most suitable, most effective, and safest drug or treatment, but also the “tailor-made” dosage.

Pediatric therapies usually rely on previous clinical experience in adults. However, there exists scientific evidence that drug pharmacokinetics and pharmacodynamics in children differ from those in adults. For example, the interaction of specific drugs with their target receptors undergoes changes over the maturation of the different organs and systems. A similar phenomenon is observed for toxicity and adverse effects. Thus, it is clear that the treatment of disease in children cannot be simplified to the direct adjustment of the dose to the body weight/surface [1]. 

Children cannot be considered “miniature adults”. There are important anatomical, physiological, and developmental differences in the various organs between these two populations, including between different pediatric age groups. The ways in which the body and drugs interact with each other (pharmacokinetics and pharmacodynamics) change, particularly comparing infants (even more so if premature) and adults. Differences in the absorption, metabolism, distribution, and elimination of drugs can cause the so-called half-life (a pharmacokinetic indicator expressing the time interval required for the plasma concentration of the drug to decrease by 50 percent) in the newborn to be about twice that observed in the adult. This means that at the same dose normalized for body weight, the concentration of the drug in the infant decreases more slowly than in the adult [2]. 

For this reason, drugs should be properly studied in the pediatric population before they are administered, which, even today, despite advances, is still not guaranteed.

Over the past fifty years, pharmaceutical research has made great progress in the area of treatment for adults, but the same cannot be said in the case of pediatric medicines. About 50 percent of pediatric medicines, as mentioned above, are administered without first being tested on children. While it is true that the new figures attest to some progress, many medicines continue to be used “off-label” [3], while others are even used unlicensed, that is, without authorization. Overall, it appears that the use of medicaments for pediatric use is an area that has so far not been sufficiently studied and is worthy of further investigation, and that a large number of medicaments newly introduced to the market are not tested in the various pediatric age groups. This fact led Harry Shirkey to use as early as the late 1960s the definition, referring to children, of therapeutic orphans [4]. Regarding the side effects of medicines, the absence of pediatric authorization poses a higher risk for children than for adults.

In this Special Issue, titled “Recent Advances in Therapeutic Strategies for the Treatment of Pediatric Diseases”, we proposed to collect original research reports, reviews, and perspectives regarding the treatment of pediatric diseases. In fact, although to date several pediatric diseases are efficiently treated, any efforts in the development of new therapeutic strategies are appreciated to reduce side effects and ameliorate the life styles of affected children.

The Special Issue consists of eight published papers (four research articles, three review articles, and one systematic review) presenting different and significant advances in this field.

In contribution 1, Adamiszak and colleagues evaluated fluconazole dosing regimens for Candida spp. prophylaxis in hemato-oncologic pediatric patients. Although the results shown require clinical validation by assessing a larger number of patients, the authors conclude that hemato-oncologic pediatric patients require increased fluconazole doses to obtain the therapeutic efficacy.

Zuccari et al. (contribution 2) investigated the colloidal stability of the liposomes and the chemical stability of amphotericin B over time at storage conditions, demonstrating that the most diluted formulation (addressed to the more vulnerable patients), kept at room temperature, showed marked changes in the aggregation state and high cytotoxicity. For these results, the authors concluded that the centralization of the dilution of AmBisome^®^ at the pharmacy in the G. Gaslini children hospital is extremely relevant for patient safety.

It is known that oral forms (capsules) of drugs are not adapted for use in children. However, in contribution 3 Caroline Lemarchand and colleagues demonstrated that mixing Temozolomide (TMZ, a drug used in managing pediatric cancers) capsule content with food may result in significant underexposure, as it is likely that children will not complete their meals. The study presented in contribution 4 showed that oral TMZ suspension and TMZ oral capsule treatment are bioequivalent medicines without unexpected safety signals or local toxicity.

Heart disease represents the main cause of death in children with cancer due to the cardiotoxicity induced by chemotherapy treatments. So, the early detection of the cardiac damage is crucial. In contribution 5, the authors reviewed the main biomarkers for the monitoring of chemotherapy-induced cardiotoxicity, highlighting the importance of the high-sensitivity method for the detection of cardiac troponin to further improve the effectiveness of risk stratification and monitoring of cardiotoxicity during chemotherapy treatment cycles.

As mentioned above, many drugs never tested on children are prescribed by pediatricians. In contribution 6, authors examined in a comprehensive review the prevalence of off-label drug use in pediatrics by conducting a literature search and including studies from 2012 to 2022 that assessed the prevalence of off-label medications in various pediatric patient populations.

Gene therapies offer incredible potential. In contribution 7 authors provided background for a recent trial exploring the use of a fixed-dose onasemnogene abeparvovec (a self-complementary AAV serotype 9 vector that encodes a functional copy of SMN1, whose mutation is responsible for spinal muscular atrophy) in children up to 5 years of age through intrathecal administration. Marco Zaffanelli and colleagues (contribution 8) presented a systematic review in which clinical evidence on the efficacy and safety of intranasal corticosteroids and oral montelukast was evaluated for treating sleep-disordered breathing and adenoid hypertrophy. They concluded that the combination of intranasal corticosteroids and oral montelukast effectively and safely treats adenoid hypertrophy and mild-to-moderate OSA symptoms in children, although larger prospective trials with standardized protocols are necessary to provide robust conclusions.

There are numerous difficulties underlying the small number of pediatric trials, which can be summarily identified as organizational–managerial, economic, and ethical.

From the organizational point of view, pediatric trials have a greater complexity, also considering the need to minimize all procedures that involve stress and pain for the subject.

Fortunately, children in most cases represent a healthy population that mainly needs a limited number of drugs (painkillers/antifebriles, antibiotics, and respirators) for acute illnesses that resolve in a few days, such as airway infections, and pharmaceutical spending concerning children and adolescents. The poor economic return, therefore, does not encourage pharmaceutical industries from studying drugs with pediatric indication, particularly if the drug is already on the pharmaceutical market for adults.

From an ethical point of view, children are not able to choose for themselves whether to participate in a trial, and consent must be provided by parents. The fact that a child, especially a young child, will receive treatment that could cause as yet unknown side effects and be subjected to invasive or painful procedures as part of the study may be of concern to parents and a brake on participation.

On the other hand, however, using drugs that have not been properly designed for that age group could pose greater health risks to the child than a clinical trial.

In conclusion, the landscape of pediatrics is rapidly evolving due to continued advances in genetics, biological therapies, and the understanding of inflammatory and infectious diseases. These advances not only improve our ability to treat rare and complex diseases but also offer new hope and possibilities to our youngest patients and their families. In the future, the further integration of biotechnology with daily clinical practice promises to radically transform pediatrics, leading to increasingly personalized care based on the individual needs of the child.

## References

[1] Alrasheed M.M., Alakeel Y.S., Yelbuz T.M., Bin-Moallim M.A., Husain W.J.M., Alakeel Y.S., Kabbani M.S., Alghamdi A.A. (2024). Developmental Pharmacokinetics. Manual of Pediatric Cardiac Care.

[2] Kimland E., Odlind V. (2012). Off-label drug use in pediatric patients. Clin. Pharmacol. Ther..

[3] Conroy S., Choonara I., Impicciatore P., Mohn A., Arnell H., Rane A., Knoeppel C., Seyberth H., Pandolfini C., Raffaelli M.P. (2000). Survey of unlicensed and off label drug use in paediatric wards in European countries. BMJ.

[4] Shirkey H. (1968). Therapeutic orphans. J. Pediatr..

